# The RHO-1 RhoGTPase Modulates Fertility and Multiple Behaviors in Adult *C. elegans*

**DOI:** 10.1371/journal.pone.0017265

**Published:** 2011-02-28

**Authors:** Rachel McMullan, Stephen J. Nurrish

**Affiliations:** MRC Cell Biology Unit, MRC Laboratory for Molecular Cell Biology and Department of Neuroscience, Physiology and Pharmacology, University College, London, United Kingdom; Brown University, United States of America

## Abstract

The Rho family of small GTPases are essential during early embryonic development making it difficult to study their functions in adult animals. Using inducible transgenes expressing either a constitutively active version of the single *C. elegans* Rho ortholog, RHO-1, or an inhibitor of endogenous Rho (C3 transferase), we demonstrate multiple defects caused by altering Rho signaling in adult *C. elegans*. Changes in RHO-1 signaling in cholinergic neurons affected locomotion, pharyngeal pumping and fecundity. Changes in RHO-1 signaling outside the cholinergic neurons resulted in defective defecation, ovulation, and changes in *C. elegans* body morphology. Finally both increased and decreased RHO-1 signaling in adults resulted in death within hours. The multiple post-developmental roles for Rho in *C. elegans* demonstrate that RhoA signaling pathways continue to be used post-developmentally and the resulting phenotypes provide an opportunity to further study post-developmental Rho signaling pathways using genetic screens.

## Introduction

Rho GTPases regulate many basic cell functions. Using cell based assays they have been implicated in the establishment of cell polarity, cell-shape change, cell migration, phagocytosis, secretion, cell-cycle progression, cytokinesis and transcription [1]. These studies have told us a great deal about the functions of Rho, however in some cases the importance of these functions in whole animals remains unclear. Here we investigate the role of RhoA in adult *C. elegans*.

*In vivo* studies involving Rho, its regulators or effectors have largely focused on their many developmental roles [2]. RNAi of the single RhoA orthlog in *C. elegans* (*rho-1*) resulted in early embryonic arrest, with a failure in cytokinesis, revealing a role for Rho signaling at the earliest stages of development [3]. Studies of Rho signaling in *C. elegans* also show that Rho is required throughout development to regulate many other processes including neuronal morphogenesis and axon pathfinding [2], [4], ventral hypodermal closure [5], gastrulation [2], [6] and vulval development [7]. The requirement for Rho signaling during development is not restricted to *C. elegans* and inactivation of RhoA, Rac1 or Cdc42 in mice or *Drosophila* results in embryonic lethality [8], [9], [10] showing that this requirement is evolutionarily conserved.

Does Rho also function post-developmentally? Although inactivation of some GTPases causes embryonic lethality inactivation of others (RhoB, RhoC, Rac2 or Rac3) results in viable, fertile adult mice [10], [11], [12], [13], [14]. In some cases defects can be observed in these adult animals for example mice lacking the haematopoetic-cell-specific GTPase Rac2 or conditionally lacking Rac1 have impaired adult immune function [15]. A naturally occurring dominant negative mutation in Rac2 has been found in a patient with severe recurrent infection and reduced neutrophil chemotaxis, emphasising the importance of Rac2′s function in the human immune system [16], [17]. Behavioral studies of adult mice lacking Rho regulators and effectors highlight a conserved role for Rho in the nervous system. Mice with mutations in PAK, WAVE-1, or LIMK-1 have learning and memory defects [18], [19], [20] while mutations in human genes encoding regulators (ARHGEF6, OPHN1) or effectors (LIMK-1, PAK3) are associated with mental retardation [21], [22].

Although these genetic studies have associated Rho GTPases with cellular responses and behaviors in adult animals, it is likely that at least some of the defects described above reflect subtle developmental problems rather than roles for Rho signaling in adults. Both Rac1 and Rac2 have roles in haematopoietic cell development, and it is possible that these developmental effects may contribute to the defective immune function seen in adult mice deficient in these proteins [23]. To study the post-developmental functions of Rho GTPases it is necessary to exclude such developmental roles.

Using *C. elegans* we have previously identified an adult requirement for RHO-1 signaling in synaptic function [24]. Using transgenic animals expressing a heat shock-inducible constitutively active *hsRHO-1*(*G14V*) to increase RHO-1 activity post-developmentally, we showed that RHO-1 altered locomotion behavior and neurotransmitter release in adult animals. These alterations in synaptic function were distinct from the developmental effects of RHO-1 as no changes in synapse number or gross nervous system morphology were observed [24].

We show here for the first time that, in addition to its previously published role in locomotion [24], RHO-1 acts in the cholinergic motor neurons to regulate pharyngeal pumping and fecundity. In addition, we show that RHO-1 acts in non-neuronal cells to regulate defecation and ovulation. Moreover, heat shock expression of RHO-1 in adult *C. elegans* causes a tail swelling phenotype similar to that seen during the innate immune response to certain bacterial infections (RM and SN paper in preparation) [25], as well as a protruding vulva. Thus the use of transgenic *C. elegans* to alter RHO-1 activity allows us to study the post-developmental roles of Rho in a genetic model organism, providing an attractive system to further investigating Rho signaling using genetic screens.

## Materials and Methods

### Strains

N2 (wild type) strain was obtained from the *Caenorhabditis* Genetics Centre (University of Minnesota). *rho-1*(*ok2418*) mutant animals were generated by the *C. elegans* Gene Knockout Consortium. All strains were cultivated at 20°C unless otherwise stated and were maintained as described previously [26].

### Transgenes and germline transformation

RHO-1 and C3 transferase were as described previously (*nzIs1*, *nzEx4* and *nzEx95*) [24]. In addition activated *RHO-1*(*G14V*) expressed from the *unc-17* cholinergic promoter (QT*#*220) was injected into wild type animals at 1 ng/µl. *nzIs33* and *nzIs34* contain integrated versions of this with *p.unc-17::GFP* as an injection marker. *nzIs29* contains an integrated version with *unc-122::GFP* (a gift of P. Sengupta Brandeis University MA) as an injection marker. The neuronal phenotype of *nzIs29* phenocopies the previously described *nzIs28* transgene [24]. *hsRHO-1*(*G14V*) (QT#42) was injected into wild type animals at 1 ng/µl and *nzEx485* contains an extrachromasomal version of QT#42. *hsC3 transferase* (QT#99) was injected into wild type animals at 1 ng/µl and *nzEx5* contains an extrachromasomal version of QT#99. In all cases the data presented were obtained using *nzIs1*, *nzEx4* and *nzIs29* but similar results for locomotion, pharyngeal pumping, defecation and egg laying were obtained using at least one other independent transgenic array. *nzIs1*, *nzEx4*, *nzEx95* and *nzIs29* were used for ovulation and brood size assays.

### Induction of heat shock-inducible transgenes

Expression from the heat shock promoter was achieved using two rounds of heat shock for 60 min at 27°C or 33°C, separated by 30 min at 20°C. Animals were allowed to recover at 20°C for 30 min, 24 hours or 48 hours. Unless otherwise stated animals were heat shocked as one-day-old adults.

### Phenotypic analysis

#### Analysis of locomotion behavior

Movies showing the altered locomotion phenotype of animals expressing RHO-1(G14V) or C3 transferase were taken using a Leica M50 steromicroscope and Hamamatsu Orca-05 camera. Movies were captured at 10 frames per second for two minutes using Micromanager open source microscopy software (http://www.micro-manager.org/) and ImageJ (NIH).

#### Determination of death

Dead animals were defined as those completely lacking movement, pharyngeal pumping, defecation and egg-laying. After induction of *hsRHO-1*(*G14V*) or *hsC3transferase* we observed decaying dead animals (or ghosts) in most cases. These plates contained no animals one week after heat shock. At least 100 plates of heat-shocked *hsRHO-1*(*G14V*) or *hsC3transferase* animals have been observed.

#### Determination of pharyngeal pumping rate

Pharyngeal pumping rate was determined as described previously [27]. Rates were counted over a two minute period and averaged to give pumps/min. To assess the role of exaggerated ACh signaling in pumping animals were exposed to 1 mM aldicarb for one hour [28], prior to counting. At least 15 animals were tested in all cases.

#### Determination of defecation cycle length

The defecation cycle length was determined as described previously [29]. One-day-old adults were assayed by recording the time from one posterior body wall contraction (pBOC) to the next using Ethotimer (J.H. Thomas, University of Washington, Seattle). 10 cycles were recorded except in the case of animals with extended cycle times that were scored for 10 minutes. At least 5 animals were scored in all cases.

#### Determination of brood size

Brood size was determined using standard methods [30]. L4 stage animals were heat shocked as described above. Subsequently, individual animals of each genotype were transferred to new Nematode Growth Media (NGM) plates each day for four days and F1 progeny that reached adulthood were scored. A minimum of 8 animals was tested in all cases.

#### Determination of egg laying rate

The rate of egg laying was determined as described previously [31]. Firstly, the number of fertilized eggs remaining in adult animals was scored. One-day-old adults were placed into a solution of 1% sodium hypochlorite in M9 buffer to dissolve the adults leaving fertilized eggs that were counted using a Leica M50 stereomicroscope. At least 37 animals were tested in all cases. Secondly, the developmental stage of newly laid eggs was determined [32]. 10 one-day-old adults were placed onto a seeded NGM plate for one hour and the developmental stage of laid eggs was scored on a Zeiss Akioskop microscope. Eggs were classified into the following categories; one to two cell, three to four cell, four to eight cell, nine cell to comma stage and post-comma. Experiments were repeated four times and the data presented is the sum of these experiments.

#### Ovulation

Following heat shock adult worms were paralysed using 0.1% Tricane and 0.01% Tetramisole in M9 for 30 minutes and mounted on agarose pads. Ovulation events were recorded using a Leica DMIRB microscope with Leica 40x objective and a Hamumatsu camera. Images were taken every 3 seconds for 45 minutes using Openlab software (Improvision).

#### Larval Growth arrest

Larval growth arrest following heat shock was determined as described previously [27]. Adult animals were treated with 1% sodium hypochlorite to obtain a synchronous population of L1-staged larvae [33]. Approximately 100–200 L1 larvae were transferred to a new NGM plate and heat shocked as described above. Plates were examined 3–5 days later when wild type animals had reached adulthood. For experiments involving *nzEx4* only L1 animals containing the transgene were transferred to assay plates. Experiments were repeated at least three times.

### Statistical analysis

In all cases statistical analysis was performed using an unpaired two-tailed t-test. P values between 0.02 and 0.001 (significant) are indicated on figures using one asterisk, and P values of 0.001 or less (highly significant) are indicated with two asterisks.

## Results

### Heat shock-inducible expression of constitutively active RHO-1(G14V) can be used to model the post-developmental functions of RHO-1

To test for post-developmental roles of RHO-1 (the single *C. elegans* RhoA ortholog) we generated transgenes that expressed constitutively active RHO-1(G14V) from a heat shock-inducible promoter (*hsRHO-1*(*G14V*)*; nzIs1* and *nzEx485*) [24]. To inhibit endogenous RHO-1 we expressed the *Clostridium-botulinum*-derived C3 transferase from the same inducible promoter (*hsC3 transferase; nzEx4* and *nzEx5*). This system allowed animals to develop in the presence of normal levels of RHO-1 signaling, before we either increased (*hsRHO-1*(*G14V*)), or decreased (*hsC3 transferase*) RHO-1 signaling in adults using heat shock.

**Figure 1:**
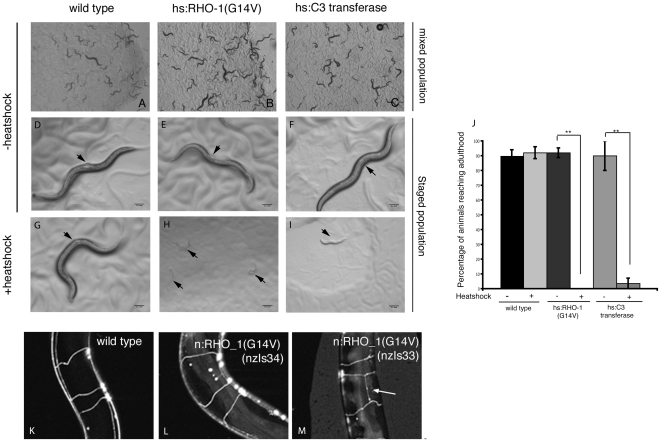
Heat shock-inducible RHO-1 is a model for post-developmental Rho signaling. (A–C). In the absence of heat shock mixed stage populations of *nzIs1* (B) or *nzEx4* (C) animals were indistinguishable from wild type (A) and were viable and fertile. (D–J). Approximately 200 L1 stage animals were transferred to assay plates and either, heat shocked immediately at 33°C, or maintained at 20°C as controls. For *hsC3 transferase* (*nzEx4*) assays 50 L1 animals carrying the GFP injection marker were selected. Following heat shock animals were maintained at 20°C and animals that had reached adulthood after 5 days were visualized. No adult animals could be observed when *hsRHO-1*(*G14V*) (*nzIs1*) (H and J) or *hsC3 transferase* (*nzEx4*) (I and J) were expressed at the L1 stage indicating that expression of these transgenes led to developmental arrest. Arrested animals are indicated with arrows in H and I. Adult animals could clearly be observed after 3 days in the absence of heat shock (D–F and J) or in wild type controls (G and J). Vulva of adult animals indicated with an arrowhead in D–G. (K, L and M). The cholinergic motor neurons of wild type animals (J) or animals expressing neuronal RHO-1(G14V) (*nzIs34* and *nzIs33*) (K and L) were labeled with *p.unc-17::gfp*. In some transgenic lines (*nzIs33*) (M) the commissures that normally extend from the ventral to the dorsal nerve cord branched prematurely extending a process towards the posterior of the animal (arrow) indicating that expression of *nRHO-1*(*G14V*) results in neuronal pathfinding defects.

Mutations that either increase or decrease RHO-1 signaling result in lethality [3]. In support of this we found that a recently isolated strain carrying a deletion in *rho-1* (*rho-1*(*ok2418*)) is homozygous lethal (data not shown). To confirm that our transgenic animals were a good model for the post-developmental effects of RHO-1 we looked to see if altering RHO-1 signaling in adults using our heat shock-inducible transgenes could bypass this lethality. In the absence of heat shock animals that carried transgenes expressing constitutively active RHO-1(G14V) or the RHO-1 inhibitor C3 transferase from the heat shock, or cholinergic specific *unc-17* promoter, were viable, fertile adults demonstrating that RHO-1 signaling in these animals was not changed sufficiently to cause developmental lethality (Figure 1A–C).

To confirm that altering RHO-1 signaling using these transgenes could induce developmental arrest and phenocopy the previously observed role for Rho signaling in development we heat shocked *hsRHO-1*(*G14V*) and *hsC3 transferase* animals during development. In contrast to wild type controls, altering RHO-1 signaling in L1 stage animals using heat shock expression of *hsRHO-1*(*G14V*) or *hsC3 transferase* was sufficient to induce developmental arrest and these animals did not become adults (Figure 1D-J).

Normal levels of RHO-1 signaling are required for correct neuronal morphogenesis [34] and expression of constitutively active Rho in cultured neurons results in defects in dendrite length and neurite outgrowth [35], [36]. Therefore we expected that overexpression of RHO-1(G14V) during development would result in defects in neuronal connectivity. Using a GFP reporter to label the motor neurons of animals expressing *RHO-1*(*G14V*) from the cholinergic motor neuron specific *unc-17* promoter (*nRHO-1*(*G14V*)) we observed premature branching of neuronal commisures in 49.0% of animals from one transgenic line (*nzIs33*) (Figure 1M). Other lines (*nzIs34*, *nzIs28* and *nzIs29*) displayed altered locomotion behavior but did not show any defects in axonal pathfinding (Figure 1K–M (*nzIs34*)) or synapse number (*nzIs28* and *nzIs29*) [24]. Thus in some cases over-expression of our activated RHO-1(G14V) transgenes caused defects in neuronal development but these defects did not correlate with the altered locomotion behavior we have described previously [24]. As we were interested in the adult neuronal function of RHO-1 we used *nzIs29* and *nzIs34* for further experiments and also sought to confirm that when RHO-1 signaling was altered in adult animals using heat shock there were no defects in neuronal connectivity. No gross defects were observed in GFP labeled motor neurons of animals that had been heat shocked as adults to induce *hsRHO-1*(*G14V*) expression post-developmentally [24]. The failure to observe axon guidance defects in *hsRHO-1*(*G14V*) adults further confirmed that these animals bypassed the essential developmental roles of RHO-1 and allow us to use these transgenes to examine the effect of changing RHO-1 signaling in adults.

### Altered RHO-1 signaling leads to behavioral changes, followed by death

Using the transgenic strains described above, we analysed the effect on *C. elegans* behavior of increasing or decreasing RHO-1 signaling post-developmentally. After heat shock adults containing the *hsRHO-1*(*G14V*) or *hsC3 transferase* transgenes displayed significant changes in locomotion behavior compared to wild type controls [24]. These locomotion changes were similar to those observed in animals expressing *RHO-1*(*G14V*) or *C3 transferase* in the cholinergic motor neurons [24]. However, 24 hours after heat shock animals carrying the *hsRHO-1*(*G14V*) transgene failed to move and the phenotypes of both *hsRHO-1*(*G14V*) and *hsC3 transferase* animals were no longer comparable to their neuronal counterparts (Movie S1–4) or wild type animals. 48 hours after heat shock 68.7% (±7.7) of *hsRHO-1*(*G14V*) animals, 58.0% (±3.6) of *hsC3 transferase* animals and 1% (±1) of wild type animals failed to show any activity (locomotion, pharyngeal pumping, egg laying, or defecation) and were scored as dead. Before death (between 30 minutes and 24 hours after heat shock), adult animals expressing either *hsRHO-1*(*G14V*) or *hsC3 transferase* displayed several phenotypes in addition to the previously described changes in locomotion [24] suggesting that altered RHO-1 signaling has multiple effects in adults.

### RHO-1 regulates pharyngeal pumping

Acetylcholine (ACh) acts as a neurotransmitter at *C. elegans* and mammalian neuromuscular junctions [37]. In *C. elegans* regulation of ACh release can alter several behaviors including pharyngeal pumping, egg laying and locomotion [38], [39], [40]. We have previously shown that RHO-1 can control ACh release and modulate locomotion behavior [24]. To further extend these findings we asked whether altering RHO-1 activity affected two other ACh-regulated behaviors; pharyngeal pumping and egg laying.

**Figure 2:**
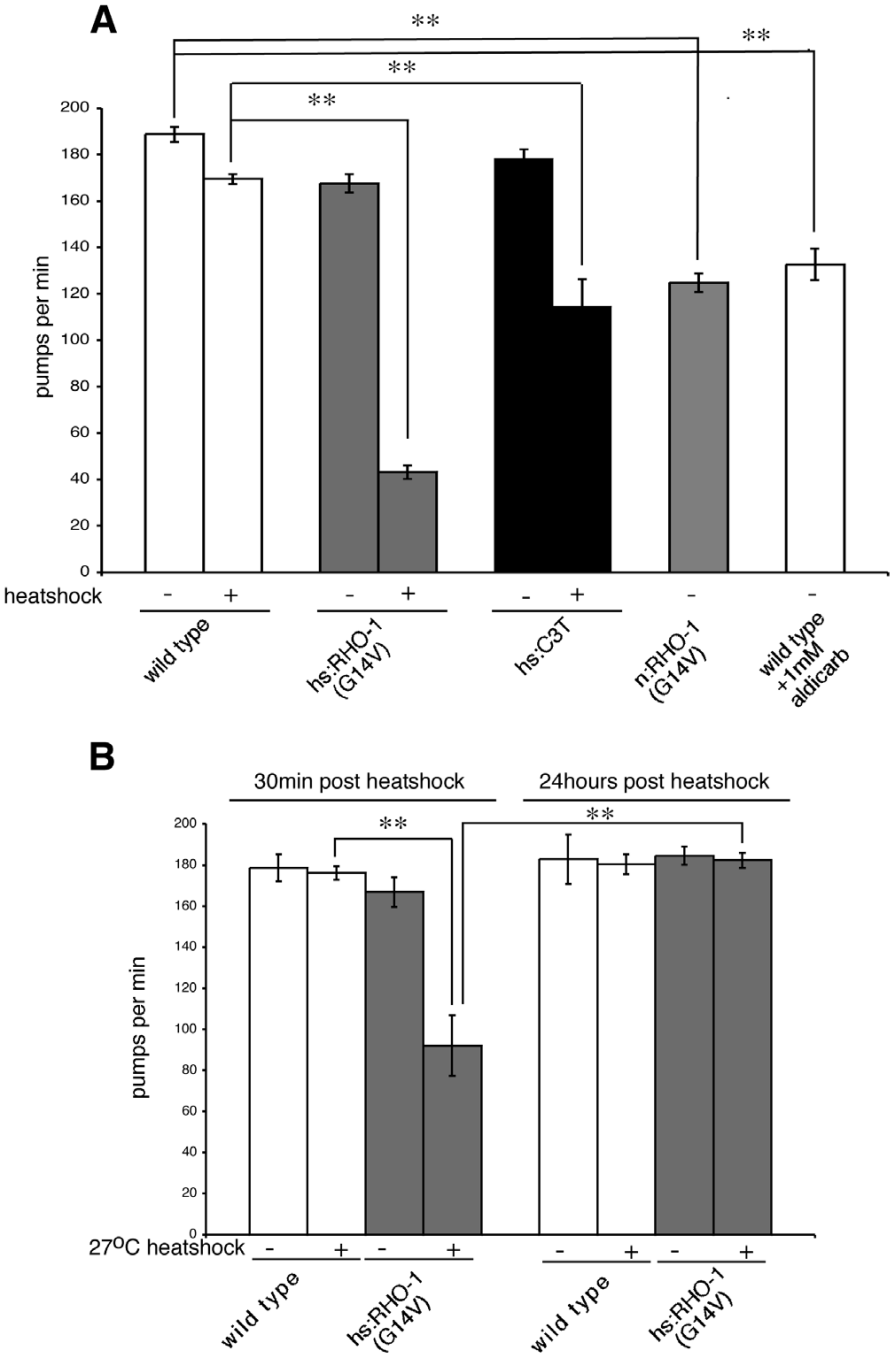
RHO-1 regulates pharyngeal pumping. Pumping rate was determined by manually counting contractions of the pharyngeal bulb for 2 min. (A) Following heat shock at 33°C animals were allowed to recover for 30 min before determining pumping rate. Expression of a constitutively activated *hsRHO-1*(*G14V*)(*nzIs1*) in adult animals or inhibition of endogenous RHO-1 using *hsC3 transferase* (*nzEx4*) decreased pumping rate. The pumping rate of animals expressing *nRHO-1*(*G14V*) (*nzIs29*) was also decreased when compared to wild type. This decrease was mimicked by increasing the extracellular ACh concentration using a 1 hour exposure to the acetylcholinesterase inhibitor aldicarb. (B) The pumping rate of *hsRHO-1*(*G14V*) (*nzIs1*) expressing animals heat shocked at 27°C was determined 30 min and 24 hours following heat shock. Expression of *hsRHO-1*(*G14V*) (*nzIs1*) resulted in a decrease in pumping rate 30 min after heat shock that was not observed after 24 hours recovery.

Expression of *hsRHO-1*(*G14V*) in adult animals decreased the rate of pharyngeal pumping even in the presence of food (Figure 2A). This decrease was observed when animals were heat shocked using our standard heat shock temperature of 33°C. A smaller, but significant, decrease was also observed when animals were heat shocked at 27°C (Figure 2B). Unlike heat shock at 33°C, a 27°C heat shock did not alter responsiveness to the ACh esterase inhibitor, aldicarb (data not shown). There are a number of possible explanations for this observation; Firstly, the pharyngeal neurons may be more sensitive to either heat shock or RHO-1(G14V) expression. Secondly, the pharyngeal muscle is more sensitive to ACh than the body wall muscle, or finally low levels RHO-1(G14V) may alter the release of something other than ACh.

Between 24 and 48 hours after heat shock at 33°C *hsRHO-1*(*G14V*) expressing animals die preventing the measurement of pumping rates at longer timepoints. However, animals heat shocked at 27°C did not die, developed normally and remained viable and fertile (data not shown). Thus the observed reduction in pharyngeal pumping rates occurred not because these animals were dying but rather as a direct consequence of altering RHO-1 signaling. In support of this we observed that 24 hours after heat shock at 27°C the rate of pumping in *hsRHO-1*(*G14V*) animals had recovered to wild type levels (Figure 2B).

The defects we observed in pharyngeal pumping could be due to expression of RHO-1(G14V) in either the neurons that control pharyngeal pumping, the pharyngeal muscle itself, or both tissues. Rho signaling has been implicated in control of both muscle contraction and release of neurotransmitter [24], [41]. We have previously shown that expression of *RHO-1*(*G14V*) from the cholinergic motor neuron specific promoter, *p.unc-17* (*nRHO-1*(*G14V*)) alters locomotion behavior and increases ACh release onto the bodywall muscles [24]. Pharyngeal pumping was decreased from 188 pumps per min in wild type animals to 124 pumps per min in *nRHO-1*(*G14V*) animals (Figure 2A) showing that altering RHO-1 activity in cholinergic motor neurons was also sufficient to regulate pharyngeal pumping. Increasing levels of extracellular ACh by briefly exposing wild type animals to the acetylcholinesterase inhibitor aldicarb also decreased pharyngeal pumping to a level similar to that observed in *nRHO-1*(*G14V*) animals (Figure 2A). These results are consistent with RHO-1 acting in the cholinergic neurons to increase ACh release and regulate pharyngeal pumping but do not exclude a role for RHO-1 in the pharyngeal muscle.

We also tested the effect of inhibiting the function of endogenous RHO-1 in adult animals using C3 transferase. Expression of *hsC3 transferase* in adult animals using heat shock at 33°C (but not 27°C, data not shown) decreased the pharyngeal pumping rate (Figure 2A); the same effect as expression of constitutively active RHO-1(G14V).

### RHO-1 regulates fecundity

**Figure 3:**
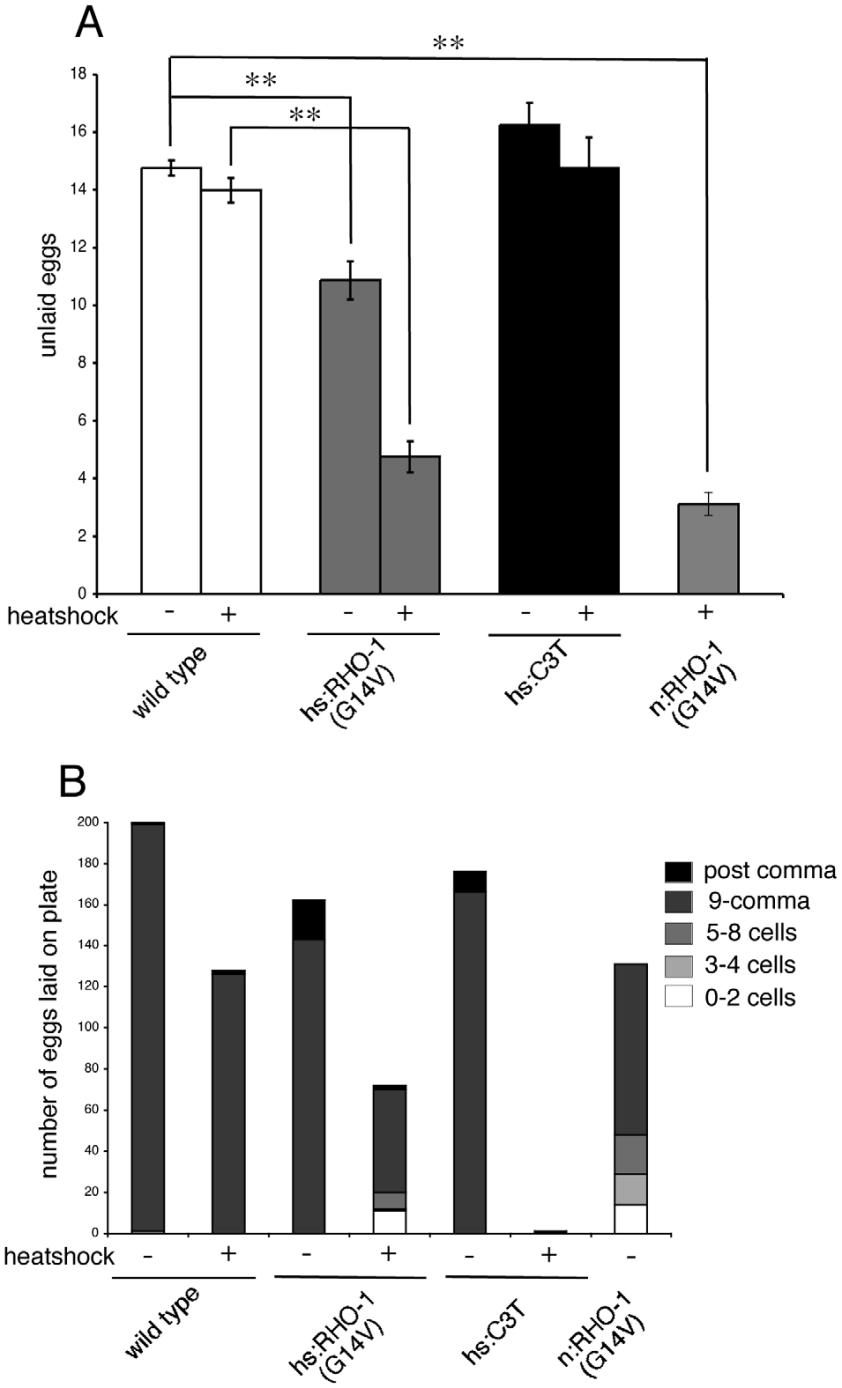
RHO-1 regulates fecundity. (A) The number of unlaid eggs remaining in animals was determined as a steady-state measure of egg laying. Expression of *hsRHO-1*(*G14V*) (*nzIs1*) or *nRHO-1*(*G14V*) (*nzIs29*) led to a significant decrease in the number of eggs remaining in animals when compared to wild type controls. Inhibition of endogenous RHO-1 using *hsC3 transferase* (*nzEx4*) did not have any significant effect on the number of eggs remaining in animals. (B) The stage of eggs laid was determined by visual inspection. Experiments were repeated four times and the data presented is the sum of these experiments. Animals expressing activated *hsRHO-1*(*G14V*) (*nzIs1*) or *nRHO-1*(*G14V*) (*nzIs29*) laid some early stage eggs containing fewer than eight cells and *hsRHO-1*(*G14V*) (*nzIs1*) the total number of eggs laid was decreased. This decrease was also observed in animals expressing *hsC3 transferase* (*nzEx4*).

Two sets of neurons (VC and HSN) control egg laying in *C. elegans* [42]. Both release ACh and serotonin; serotonin stimulates egg laying [32] whereas ACh has both positive and negative effects [40], [43]. To investigate the role of RHO-1 in this neuronally regulated behavior we tested the ability of RHO-1 to alter egg laying using two methods. We determined the number of unlaid eggs remaining in animals as a steady state measure of egg laying; animals with increased egg laying rates should retain fewer eggs than wild type while those with decreased egg laying should retain more. Even in the absence of heat shock *hsRHO-1*(*G14V*) animals showed a decrease in the number of eggs remaining in the animals suggesting that our heat shock-inducible transgenes were leaky in some cells under certain conditions (Figure 3A [44]). Expression of *hsRHO-1*(*G14V*) in adults decreased the number of eggs remaining in animals relative to heat shocked wild type and non-heat shocked *hsRHO-1*(*G14V*) animals (Figure 3A).

To further investigate RHO-1's role in egg laying we examined the number and stage of the eggs laid. Wild type animals lay eggs once they reach the 50 to 100-cell stage whereas animals with higher egg laying rates lay early stage eggs containing eight or fewer cells [32]. In contrast to wild type, animals expressing *hsRHO-1*(*G14V*) laid early stage eggs with less than eight cells (Figure 3B) suggesting that RHO-1 increases egg laying rate, however it is also possible that eggs develop more slowly in *hsRHO-1*(*G14V*) animals than in wild type. Interestingly, *hsRHO-1*(*G14V*) expressing animals also laid fewer eggs than wild type (Figure 3B) suggesting that the decreased number of eggs remaining inside *hsRHO-1*(*G14V*) mothers following heat shock is likely caused by a combination of increased egg laying and reduced egg production.

Egg laying requires motor neurons, egg-laying muscles and a supply of fertilized eggs. To address whether RHO-1 was acting in the cholinergic motor neurons to control egg laying we again used animals expressing *nRHO-1*(*G14V*). These animals laid early stage eggs and the number of unlaid eggs was decreased (Figure 3A and B) suggesting that RHO-1 signaling in the motor neurons can control both egg laying and egg production. Although *nRHO-1*(*G14V*) expressing animals laid fewer eggs than wild type they laid more eggs than *hsRHO-1*(*G14V*) expressing animals suggesting that Rho signaling may also act in non-cholinergic cells to regulate egg production (Figure 3B).

We also investigated the effect of inhibiting endogenous RHO-1 on egg laying behavior by expressing *hsC3 transferase* in adults. We observed that the number of eggs laid was decreased even in the absence of heat shock, again suggesting leaky expression from our heat shock promoter (Figure 3B [24]). However, in contrast to overexpression of *hsRHO- 1*(*G14V*), inhibition of endogenous RHO-1 did not have a significant effect on the number of eggs remaining in animals (Figure 3A). Although *hsC3 transferase* expressing animals did not retain more eggs than wild type these animals laid almost no eggs suggesting that inhibition of RHO-1 leads to decreased egg laying (Figure 3A and B). The failure to observe an increase in retained eggs despite decreased egg laying may suggest that these animals had stopped producing eggs and 24 hours after heat shock we were still unable to observe eggs accumulating inside *hsC3 transferase* expressing animals.

### RHO-1 regulates defecation

**Figure 4:**
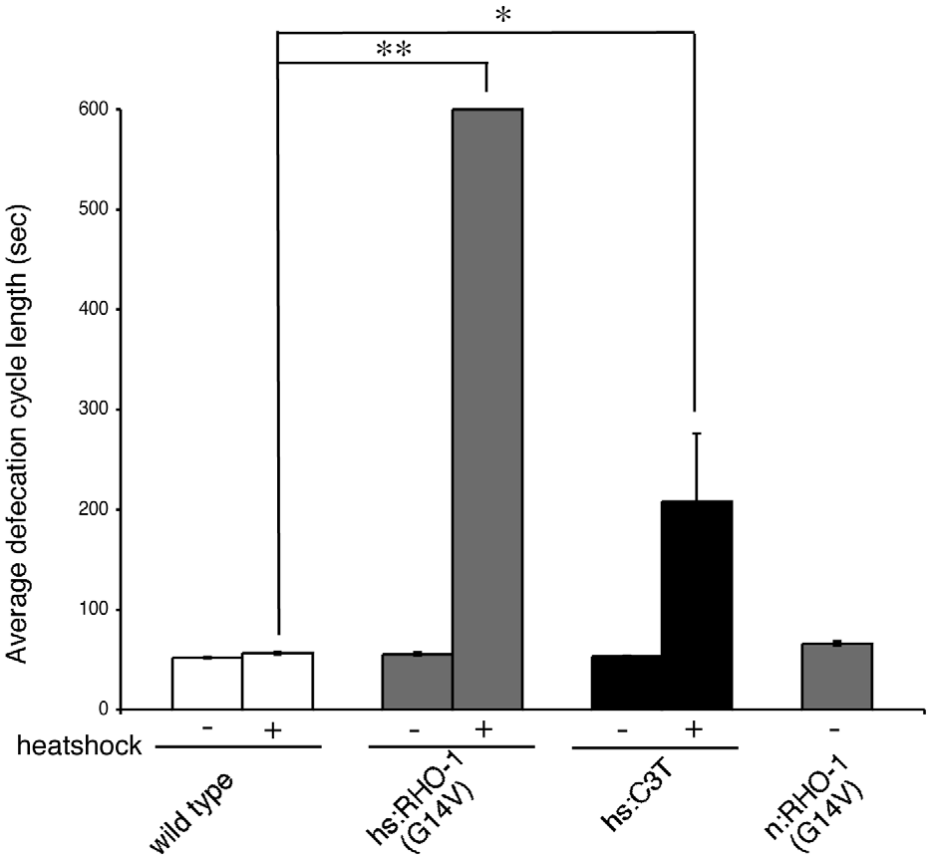
RHO-1 regulates defecation. The average defecation cycle length was determined by measuring the interval between pBoc contractions. The defecation cycle was almost completely blocked by expression of *hsRHO-1*(*G14V*) (*nzIs1*) but not by *nRHO-1*(*G14V*) (*nzIs29*). Inhibition of endogenous RHO-1 by expressing *hsC3 transferase* (*nzEx4*) resulted in increased variability in cycle length and an increase in the average defecation cycle period.

Comparison of heat shock and cholinergic expression of *RHO-1*(*G14V*) revealed roles for RHO-1 in cells other than the cholinergic motor neurons. One example is the effect of *RHO-1*(*G14V*) expression on the defecation cycle. Wild type animals defecate by initiating a series of three muscle contractions; (posterior body-wall-muscle contraction (pBoc), anterior body-wall-muscle contraction (aBoc) and enteric-muscle contraction (Emc)), regularly with an interval between cycles of 50sec [29]. Although the aBoc and Emc steps of this cycle require neuronal activity via a GABAergic motor neuron the pBoc step and initiation of the defecation cycle do not require neuronal input [45]. Expression of constitutively active *hsRHO-1*(*G14V*), but not *nRHO-1*(*G14V*) resulted in an almost complete block in initiation of the defecation cycle (as defined by the interval between pBoc) (Figure 4), however, when a cycle did occur all three motor steps (pBoc, aBoc and Emc) were observed. This suggests that RHO-1 acts in non-cholinergic cells to regulate defecation cycle initiation rather than muscle contractions themselves. Inhibition of endogenous RHO-1 in adults using *hsC3 transferase* also resulted in a defect in the defecation motor programme. Although the cycle was not completely blocked, as it was by overexpression of *hsRHO-1*(*G14V*), it became very variable and a slight increase in the average cycle period was observed (Figure 4).

### RHO-1 regulates ovulation

**Figure 5:**
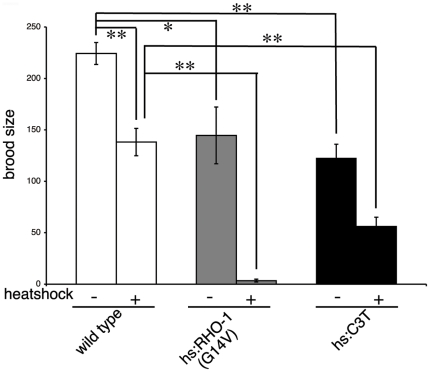
RHO-1 regulates brood size. Adult F1 progeny from animals heat shocked at the L4 stage were scored to determine brood size. Expression of activated *hsRHO-1*(*G14V*) (*nzIs1*) or *hsC3 transferase* (*nzEx4*) resulted in a decrease in brood size.

Our analysis of fecundity revealed that when RHO-1 signaling was activated in adults the total number of eggs was decreased suggesting a defect in egg production. Therefore, we asked whether RHO-1 signaling could affect oocyte fertilization. Failure to fertilize oocytes would result in a decrease in brood size and so we determined the brood size of animals expressing *hsRHO-1*(*G14V*). Although we observed a decrease in the brood size of wild type animals using our heat shock conditions we also observed that expression of activated *hsRHO-1*(*G14V*) in L4 stage animals resulted in a much larger decrease in brood size when compared to controls (Figure 5).

The decrease in brood size we observed could, in part, be caused by the premature death of the parent and the defects in development that we have described following *hsRHO-1*(*G14V*) expression. Therefore, to directly assess the effect of increased RHO-1 signaling on ovulation, we recorded ovulation events in adults expressing *hsRHO-1*(*G14V*). *C. elegans* are self-fertilizing hermaphrodites, thus, eggs can be produced by self- fertilization of oocytes as they pass through the spermatheca. This process occurs in a series of steps; oocyte maturation, dilation of the spermatheca and contraction of the sheath cells surrounding the oocyte. The contraction of the sheath cells pulls the spermatheca around the oocyte allowing entry into the spermatheca and fertilization [30]. Ovulation events in wild type animals, both before and after heat shock, appeared normal and we observed at least one ovulation event in every animal within the 45 minute-recording period (Movie S5). However, in *hsRHO-1*(*G14V*) animals without heat shock the increase in sheath cell contractions that precedes ovulation was not observed and we could not record any ovulation events during the 45 minute recording (Movie S6). As *hsRHO-1*(*G14V*) animals contain eggs and are viable and fertile ovulation must occur. It is possible that leaky expression of *hsRHO-1*(*G14V*) results in a reduction in the number of ovulation events and this may account for the decrease in brood size we observed in these animals. Heat shock induction of *hsRHO-1*(*G14V*) also resulted in no observed ovulations and in addition, we observed a large increase in sheath cell contractions that was not confined to just before oocyte entry into the spermatheca (Movie S7). *hsRHO-1*(*G14V*) animals did not ovulate because oocytes failed to exit the spermatheca towards the vulva and were occasionally pushed out of the spermatheca in the opposite direction (Movie S7). The different ovulation phenotypes observed before or after heat shock suggests that RHO-1 may have multiple effects on fertilization with low and high levels of RHO-1(G14V) activity causing different responses, however, as both phenotypes result in decreased fertilization they may account for the decrease in total egg number we observed previously. Using animals expressing *nRHO-1*(*G14V*) in the cholinergic motor neurons we detected normal ovulation events indicating that this was not the site of action for RHO-1 (data not shown).

We also asked whether inhibition of endogenous RHO-1 using *hsC3 transferase* had any effect on fertilization. In the absence of heat shock *hsC3 transferase* animals had a decreased brood size and no ovulations were detected (Figure 5 and data not shown), again suggesting leaky expression from our heat shock promoter. Increased expression of *hsC3 transferase* using heat shock further decreased the brood size (Figure 5) and consistent with this no fertilization of oocytes was observed during a 45 minute recording in adults expressing *hsC3 transferase* (Movie S8).

### Overexpression of RHO-1 causes *dar* and *pvl* phenotypes

**Figure 6:**
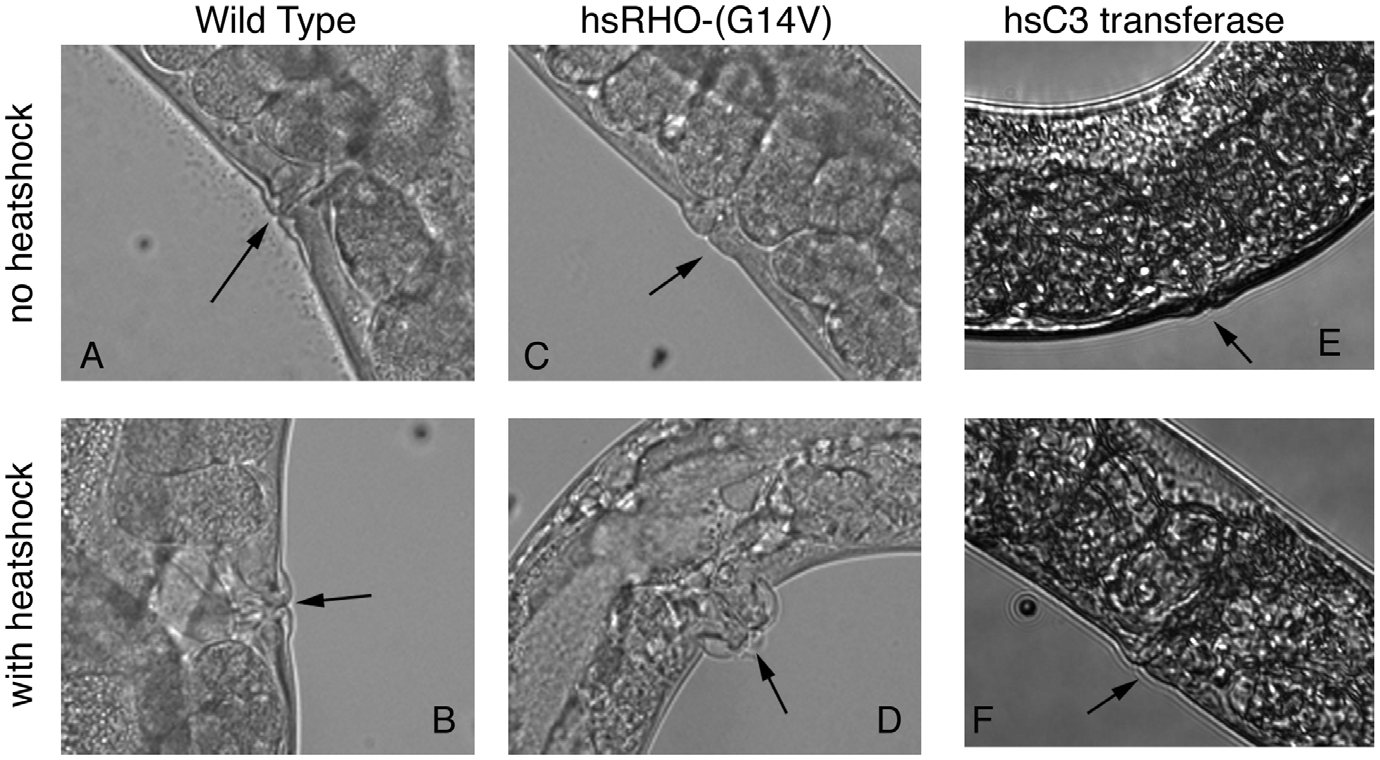
Overexpression of *hsRHO-1*(*G14V*) results in anatomical changes. After heat shock abnormalities were observed in the vulva of animals expressing *hsRHO-1*(*G14V*) (*nzIs1*). Wild type controls (A and B), animals expressing *hsC3 transferase* (*nzEx4*) (E and F) and animals containing the *hsRHO-1*(*G14V*) (*nzIs1*) transgene in the absence of heat shock (C) had normal unswollen vulva however following heat shock of *hsRHO-1*(*G14V*) (*nzIs1*) animals a phenotype reminiscent of *pvl* was observed (D).

During the course of our studies we observed changes in the anatomy of animals expressing *hsRHO-1*(*G14V*). Animals induced to express *hsRHO-1*(*G14V*) by heat shock at 33°C (Figure 6D) and then allowed to recover developed swellings around the vulval (Figure 6C and D) and anal (data not shown) regions. These swellings became visible 6 hours after heat shock, had a penetrance of 90% and 93% respectively 12 hours after heat shock and resembled the *pvl* and *dar* phenotypes previously reported [25], [46]. We further describe a role for Rho signaling in regulating the *dar* phenotype following infection elsewhere (RM and SN paper in preparation). We did not observe these phenotypes in wild type animals or animals expressing *hsC3 transferase* subjected to identical heat shock regimes (Figure 6A, B, E and F) and therefore these phenotypes appear to be a consequence of increased RHO-1 signaling.

## Discussion

In this paper we have characterised the post-developmental role of RHO-1, the RhoA ortholog in *C. elegans.* RhoA's essential functions in development make it difficult to study its role in adult animals, particularly using genetics, as animals with mutations in Rho GTPases are likely to be dead or have severe developmental defects. Indeed we observed that a deletion in the *rho-1* gene (*rho-1*(*ok2418*)) causes lethality, although rescue experiments need to be performed to confirm that the *rho-1* deletion is responsible for this lethal phenotype. Here we show that using heat shock-inducible transgenes to either increase or decrease RHO-1 activity, and control when RHO-1 signaling is altered, we can bypass the early lethality caused by too much or too little RHO-1 signaling.

Using heat shock to express constitutively active RHO-1(G14V) from our integrated transgene (*nzIs1*) in adult animals we did not observe the neuronal pathfinding defects that were observed when RHO-1(G14V) was expressed neuronally in larval animals. This demonstrates that we are able to control the timing of when RHO-1 signaling becomes aberrant however we and others have observed phenotypes that are consistent with leaky expression from the heat shock promoters of both the *hsRHO-1*(*G14V*) and *hsC3 transferase* transgenes used here [44]. Increased egg laying and decreased brood size and pharyngeal pumping were observed in *hsRHO-1*(*G14V*) animals even in the absence of heat shock, although in all cases the effect was far smaller than that observed in response to heat shock. It is possible that manipulation of the animals during these assays leads to stress that is sufficient to cause leaky expression from the heat shock promoter. For example even in the absence of heat shock animals containing the *hsRHO-1*(*G14V*) and *hsC3 transferase* transgenes failed to ovulate when mounted on agarose pads and image over a 45 minute period and yet these animals were viable and produced progeny under our standard culture conditions. Some cells appear more prone to leaky expression from the heat shock promoter or are more sensitive to small changes in RHO-1 activity. This is supported by our observation that expression from *hsRHO-1*(*G14V*) using a weak heat shock at 27°C was sufficient to alter pharyngeal pumping but not ACh release onto the bodywall muscles or locomotion behavior. Leaky expression from the *hsRHO-1*(*G14V*) transgene may only alter adult behaviors but we cannot exclude some developmental contributions to the defects we observe. However it is clear that the use of these inducible transgenes does not cause a major change in RHO- 1 activity during development as both *hsRHO-1*(*G14V*) and *hsC3 transferase* animals become viable, fertile adults in the absence of heat shock.

Using both heat shock and cholinergic specific expression of RHO-1(G14V) we have previously shown that RHO-1 controls ACh release onto the bodywall muscles and alters locomotion behavior [24]. Here we show that RHO-1 signaling can also act in the cholinergic motor neurons to regulate pharyngeal pumping and egg laying emphasizing the importance of RHO-1 function in these neurons. The increased egg laying caused by *nRHO-1*(*G14V*) is likely due to increased activity of either the HSN or VC cholinergic motor neurons that both release ACh onto the egg laying muscles causing contraction. It will be interesting to express *RHO-1*(*G14V*) in just the HSN neurons or test the effect of *nRHO-1*(*G14V*) in *egl-1* mutants, which lack the HSN neuron. Cholinergic expression of constitutively active RHO-1(G14V) decreased the rate of pharyngeal pumping and yet from our previous studies RHO-1(G14V) would be expected to increase ACh release onto the pharyngeal muscle, which should increase pumping [38]. Addition of the acetylcholinesterase inhibitor aldicarb; which increases extracellular ACh levels and causes hypercontraction of the body wall muscles [28], also decreased pumping rate after one hour and chronic exposure to aldicarb causes death due to hypercontraction of the pharyngeal muscles [47], [48]. Thus, it is likely that cholinergic expression of constitutively active RHO-1(G14V) triggers a release of ACh sufficient to cause prolonged contraction of the pharyngeal muscle and thus decrease the pumping rate. Alternatively, *nRHO-1*(*G14V*) expression in pharyngeal motor neurons may alter more than ACh release, for example neuropeptides and octopamine that can both reduce contraction of the pharyngeal muscle [49]. Similarly, in the egg laying system the HSN neuron releases serotonin and neuropeptides in addition to acetylcholine. *nRHO-1*(*G14V*) may also alter their release onto the egg laying muscles. It is possible that Rho could regulate neuropeptide release through its ability to regulate levels of the membrane-bound second-messenger DAG as the DAG binding protein PKC-1 stimulates neuropeptide release in *C. elegans* [50]. As locomotion, egg-laying, and pharyngeal pumping are all controlled by ACh it is possible that the RHO-1 pathways involved in these three behaviors [51] are substantially similar if not identical, although one or more RHO-1 effectors in the nervous system still remains to be identified [24].

We observed more phenotypes associated with *RHO-1*(*G14V*) expression from the heat shock promoter than from the cholinergic promoter suggesting that RHO-1 signaling pathways are also used outside the cholinergic neurons. Most strikingly animals that expressed *RHO-1*(*G14V*) from the widely expressed heat shock promoter became sterile and died following heat shock, whereas animals that expressed *RHO-1*(*G14V*) in the cholinergic motor neurons were viable and fertile. In addition *hsRHO-1*(*G14V*) animals were defective in their ability to initiate the defecation cycle after heat shock, had ovulation defects and developed *dar* and *pvl* phenotypes. None of these phenotypes were observed in *nRHO-1*(*G14V*) animals confirming that these phenotypes require RHO-1 signaling in cells other than the cholinergic neurons. Our data demonstrates that the expression of *RHO-1*(*G14V*) results in a pleiotrophic phenotype and it is likely that Rho signaling may be required in multiple tissues to influence some behaviors. For example heat shock and neuronal expression of RHO-1(G14V) appears to influence egg laying however decreased ovulation and slower development may contribute to the egg laying phenotype we observed. The use of recently described tools, such as the FLP/FRT system [52], that allow temporal and spatial regulation of gene expression will enable expression of RHO-1 in specific adult tissues and will begin to address Rho's role in each of these processes.

Inhibition of endogenous RHO-1 using *hsC3 transferase* also caused changes in pharyngeal pumping, egg laying, defecation cycle length, and fertility similar to those observed upon expression of *hsRHO-1*(*G14V*). This was in contrast to changes in locomotion and ACh release where RHO-1(G14V) and C3 transferase had opposite effects and the *dar* and *pvl* phenotypes where C3 transferase had no effect. How is it that both increased and decreased RHO-1 signaling had similar effects? As the heat shock promoter is widely expressed perhaps increased Rho signaling in one cell gives rise to a similar phenotype to RHO-1 inhibition in another cell. Activation and inhibition of RHO-1 using transgenes expressed from cell specific promoters as well as more careful study of Rho's regulators and effectors will help to address this question. Indeed we have recently used this approach to demonstrate that Rho signaling is not only sufficient to cause the *dar* phenotype but is also necessary for the *dar* response to infection (R. McMullan unpublished observation). It is possible that the phenotypes we observe are somewhat non-specific because these animals were dying following transgene induction. However, this seems unlikely as we have observed defects in pharyngeal pumping and a weak *dar* phenotype in animals that appear otherwise healthy following a heat shock at 27°C. Alternatively RHO-1 may need to be able to cycle between being bound to GDP and GTP to function as has previously been observed elsewhere, for example, too much and too little RHO-1 signaling resulted in similar phenotypes in macrophage chemotaxis [53] and in Drosophila neurite outgrowth [54]. In *C. elegans* gain- and loss-of-function alleles of the rac/cdc42 like GTPase *mig-2* both caused axon pathfinding defects [2].

What are the RHO-1 signaling pathways acting in adult *C. elegans*? To further understand the role of RHO-1 signaling in adult behaviors it will be necessary to define the upstream activators and downstream effectors that modulate these behaviors. Some activators and effectors of RHO-1 signaling have been studied in *C. elegans* however mutations in several, including the Rho-associated kinase *let-502* and the serum response factor (SRF) *unc-120*, are lethal [55], [56] preventing analysis of their post-developmental functions. A more successful approach may be to analyse the behavior of transgenic animals expressing mutated versions of RHO-1 that are unable to interact with subsets of effectors in mammalian systems [57]. Mutations in some Rho effectors are not lethal, for example animals with mutations in the DAG Kinase, *dgk-1*, which is negatively regulated by RHO-1, have defects in locomotion and egg laying [58] consistent with its function downstream of RHO-1. The defecation and *dar* phenotypes observed in *hsRHO-1*(*G14V*) animals have not been observed in *dgk-1* mutants and these animals become viable, fertile adults [58], [59]. Thus, RHO-1 must regulate other downstream effectors to control these behaviors.

One pathway known to interact with Rho in mammalian cells is the Ras/ERK/MAPKinase pathway [60], [61], [62]. Interestingly, both these pathways are required to regulate the *dar* phenotype in response to infection ([63] R. McMullan personal observation). We describe the interaction between these pathways in detail elsewhere and show that Rho and Ras signaling converge on the ERK/MAPKinase pathway to trigger this immune response. Previous studies of *C. elegans* body morphology and behavior have provided insights into many conserved signaling pathways such as the Ras/MAPK pathway and genetic screens have identified core components, regulators and interactions with other signaling pathways [64], [65]. These findings establish the *dar* phenotype as a genetic model to dissect interactions between Rho and ERK/MAP Kinase signaling pathways. The *pvl* phenotype has been previously observed in mutants defective for Wnt signaling although in this case it is thought to be a developmental defect [46]. Activation of RHO-1 results in a much weaker *pvl* phenotype and does not completely phenocopy the protruding vulva seen during development however, in mammalian cells, RhoA has been shown to be a component of non-canonical Wnt signaling pathways [66] raising the possibility that Wnt signaling is required to both form the vulva and maintain its morphology and Rho signaling interferes with this. Suppressor and enhancer genetic screens will reveal the pathways by which constitutively active RHO-1(G14V) causes both the *dar* and *pvl* phenotypes.

Recent work has provided clues to the nature of the upstream components of Rho signaling in adult *C. elegans*. The adult behaviors of three RhoGEF mutants, *vav-1*, *unc-73* and *rhgf-1*, have been studied. Deletion of the RhoGEF2 domain of *unc-73* that activates RHO-1 results in developmental arrest due to defects in pharyngeal pumping [67], however, animals with pharynx specific rescue of *unc-73* [67] or mutations specific to the UNC-73 RhoGEF2 domain [68] develop into adults that have lethargic locomotion and egg laying defects. Loss of *vav-1* also led to defects in pumping as well as defects in gonadal-sheath-cell contractions and defecation [30]. Both VAV-1 and UNC-73 can regulate the activity of multiple Rho family small GTPase and our results suggest that the pharyngeal pumping, defecation, and ovulation defects observed in *vav-1* and *unc-73* mutants are due to a defect in RHO-1 activation. The data obtained so far suggests that behaviors such as defecation and ovulation may be regulated by one RhoGEF (VAV-1) acting on RHO-1 however as both *unc-73* and *vav-1* appear to regulate pharyngeal pumping it seems that RhoGEFs may act redundantly to regulate RHO-1 signaling in some behaviors. In support of this, data from our laboratory suggest that, in addition to *unc-73*, *rhgf-1* is able to regulate RHO-1 activity and control locomotion [69]. These RhoGEFs are likely to be activated by different signals as *unc-73* is regulated by Gαq [68] while *rhgf-1* acts downstream of Gα12 [69]. Whilst RhoGEF mutations cause adult phenotypes it cannot be ruled out that this is a result of a developmental defect. Careful analysis of any mutants in RHO-1 signaling components will be required to distinguish the adult roles of RHO-1 signaling from the developmental ones.

Analysis of adult Rho GTPases signaling pathways is important as aberrant signaling by these pathways has been implicated in human diseases including cancer, neurological disorders, vascular and renal disease [70], [71], [72], [73]. Rho GTPase signaling has been extensively studied using biochemistry and cell based assays [74] and Rho's role in development has been confirmed in whole animal models [75]. Here we use a whole animal model to show that Rho also has important post-developmental roles acting in both neuronal and non-neuronal tissues. *C. elegans* has a single Rho GTPase, RHO- 1, and we show that aberrant RHO-1 activity alters adult processes such as neuronal activity, fertility, defecation and cell morphology and results in death. Our work provides the starting point for future genetic screens to fully describe adult RHO-1 signaling pathways.

## Supporting Information

Movie S1 **Adult** ***nRHO-1***(***G14V*** **) (** ***nzIs29*** **) animals displayed loopy locomotion compared to wildtype animals.**
(MP4)

Movie S2 **Adult** ***hsRHO-1***(***G14V*** **) (** ***nzIs1*** **) animals were heat shocked at 33°C and their locomotion behavior was recorded 24 hours after recovery at 20°C.** Although some animals displayed loopy locomotion the majority of animals only moved slightly when touched.
(MP4)

Movie S3 **Animals expressing** ***nC3 transferase*** **(** ***nzEx95*** **) show lethargic locomotion behavior when compared to wild type controls (Movie S1)** [**24**] **.**
(MP4)

Movie S4 **Adult** ***hsC3 transferase*** **(** ***nzEx4*** **) animals were heat shocked at 33°C and their locomotion behavior was recorded after recovery for 24 hours at 20°C.** Animals expressing *nC3 transferase* (*nzEx95*) or *hsC3transferase* (*nzEx4*) 30 min following heat shock ([24] and movie S4) were lethargic but still responded to touch however 24 hours after heat shock animals no longer responded strongly to touch.
(MP4)

Movie S5 **Ovulation events in wild type animals were recorded for 45minutes as described in** **Materials and Methods** **.** Wild type animal ovulate as previously described [48] showing an increase in sheath cell contractions prior to ovulation followed by entry of the oocyte into the spermatheca, fertilization and exit into the uterus. One ovulation event can be observed during this recording.
(MP4)

Movie S6 **Ovulation events in adult** ***hsRHO-1***(***G14V*** **) (** ***nzIs1*** **) animals in the absence of heat shock were recorded for 45 minutes as described in** **Materials and Methods** **.** These animals failed to ovulate during the recording period.
(MP4)

Movie S7 **Adult** ***hsRHO-1***(***G14V*** **) (** ***nzIs1*** **) animals were heat shocked at 33°C and ovulation events were recorded for 45minutes as described in** **Materials and Methods** **.** *hsRHO-1*(*G14V*) (*nzIs1*) expressing animals show increased sheath cell contractions and failure of oocytes to correctly exit the spermatheca. No complete ovulation events were observed during the recording period.
(MP4)

Movie S8 **Adult** ***hsC3 transferase*** **(** ***nzEx4*** **) expressing animals were heat shocked at 33°C and ovulation events were recorded for 45minutes as described in** **Materials and Methods** **.** *hsC3 transferase* (*nzEx4*) expressing animals showed an increase in sheath cell contractions throughout the recording period however oocytes completely failed to enter the spermatheca and no ovulation events were observed during the recording.
(MP4)
